# Accelerating the open research agenda to solve global challenges

**DOI:** 10.1002/ece3.10887

**Published:** 2024-01-31

**Authors:** Zuzanna B. Zagrodzka, Thomas F. Johnson, Andrew P. Beckerman

**Affiliations:** ^1^ Ecology and Evolutionary Biology, School of Biosciences University of Sheffield Sheffield UK

**Keywords:** communication barriers, evidence‐based policies, global challenges, knowledge mobilisation, open research

## Abstract

Harnessing science‐based policy is key to addressing global challenges like the biodiversity and climate crises. Open research principles underpin effective science‐based policy, but the uptake of these principles is likely constrained by the politicisation, commoditisation and conflicting motives of stakeholders in the research landscape. Here, using the mission and vision statements from 129 stakeholders from across the research landscape, we explore alignment in open research principles between stakeholders. We find poor alignment between stakeholders, largely focussed around journals, societies and funders, all of which have low open research language‐use. We argue that this poor alignment stifles knowledge flow within the research landscape, ultimately limiting the mobilisation of impactful science‐based policy. We offer recommendations on how the research landscape could embrace open research principles to accelerate societies' ability to solve global challenges.

## Graphical abstract

Open research principles underpin the effective science‐based policy key to tackling global challenges; however, the uptake of these principles is likely constrained by the politicisation, commoditisation and conflicting motives of stakeholders in the research landscape. Here, using the mission and vision statements from 129 stakeholders from across the research landscape, we explore alignment in open research principles. We find that poor alignment between different stakeholders stifles knowledge flow within the research landscape, ultimately limiting the mobilisation of impactful science‐based policy.

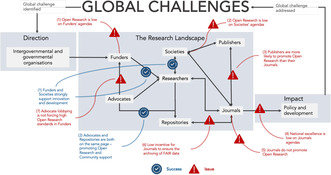

## 1 INTRODUCTION

Evidence‐based policy—the process of generating and mobilising knowledge and solutions that can support decision‐making—underpins societies' ability to address global challenges like the climate and biodiversity crises (Moyer & Hedden 2020). The open research agenda (UNESCO, 2021) is vital for such science‐based policy and has three key features: (1) open access to publications, methods and tools; (2) FAIR data (Wilkinson et al., 2016); and (3) an increase in transparency, accountability, equity and collaboration (UNESCO, 2021). These core principles mobilise vital knowledge, increase research validation (O'Dea et al., 2021; Thibault et al., 2023) and improve the robustness and accuracy of research insight (Usui et al., 2021)—increasing public trust in evidence (Voulvoulis & Burgman, 2019).

Inaccessible research and the slow mobilisation of knowledge are the norm. Rapid growth of published scientific articles and data deposited in public repositories (Heberling et al., 2021) should provide a strong platform for addressing global challenges, but the majority of the scientific literature and data remain unavailable to the public and policy‐relevant organisations (Piwowar et al., 2018). Even when made available, essential detail and data are often missing or difficult to access. Governmental and inter‐governmental policies are consequently slow to develop around the best data, methods and knowledge (Weresa et al., 2018) and critical ideas are frequently challenged without appropriate context. Even publications by institutions such as the EU's Joint Research Centre (JRC), specifically designed to dictate open research policy for the EU, see limited adoption (Weresa et al., 2018). Unfortunately, the researcher remains the typical target for increasing open research, when other players in the research landscape likely hold the key to progress.

**FIGURE 1:**
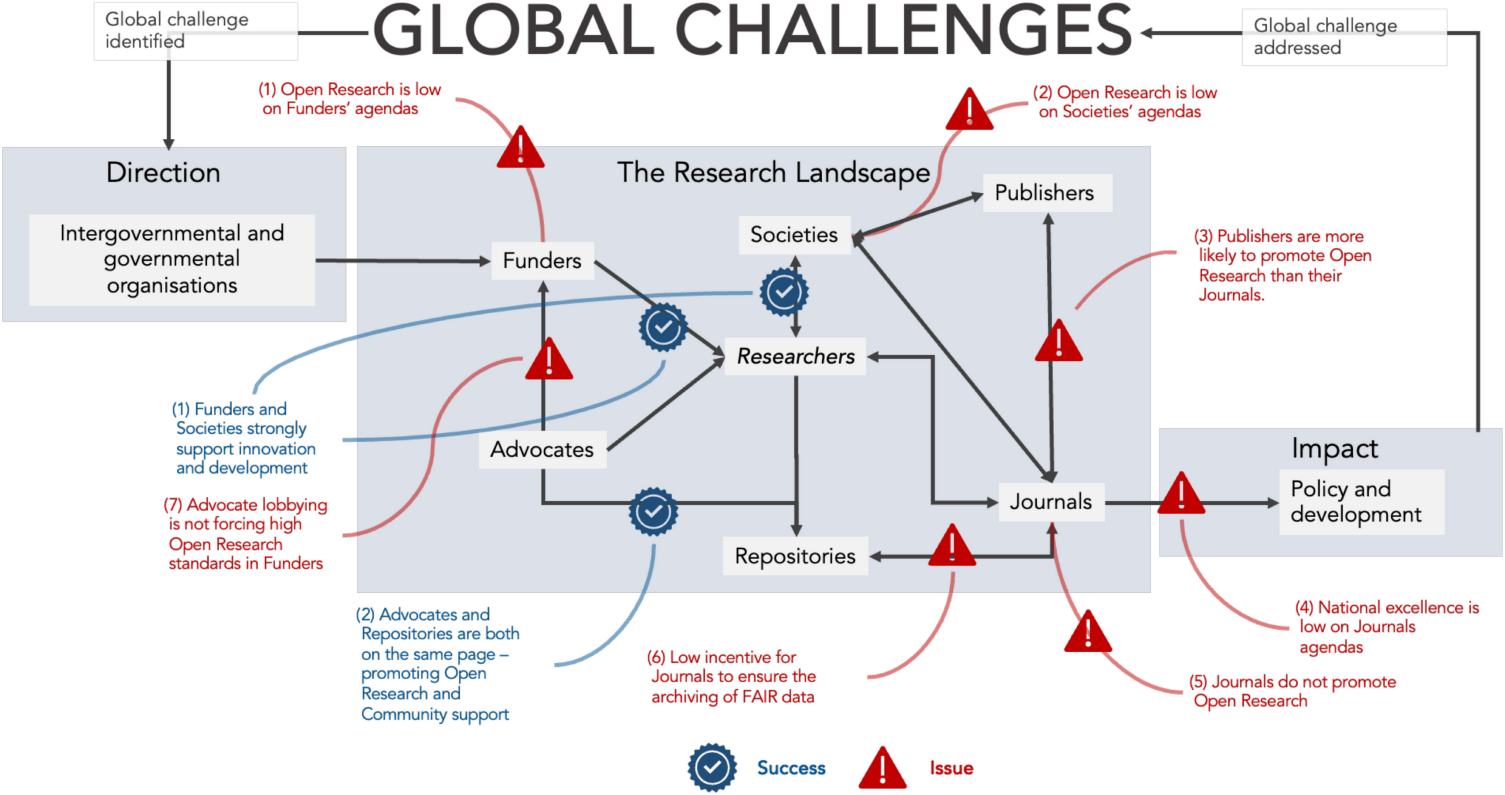
Knowledge mobilisation cycle. The research landscape is at the heart of linking policy to the solutions needed to tackle global challenges. The research landscape is characterised by several stakeholder groups; a collection of for‐profit and not‐for‐profit organisations, existing in an informal network (solid black arrows), with distinct clustering (e.g. societies, publishers and journals), which are tasked with producing and mobilising knowledge to support policy development. Against the emerging open research agenda characterised most clearly by UNESCO, there are several places where there is strong alignment with the agenda (blue ticks). However, there are far more places where one might either expect strong alignment and it does not exist or where there are clear mis‐alignments between stakeholder and open research objectives (in red). See text for methods and quantitative assessments.

A common conviction among many individuals and organisations is that the open research agenda has been stifled by the politicisation, commoditisation and conflicting motives of stakeholders in the research landscape (Figure 1). It is argued that some stakeholders operate via a business model that can lead to trade‐offs between financial decision‐making and the social and policy agendas for open research. Such trade‐offs may underpin a conflict of interest at a key point in the landscape. We focus on six stakeholder groups made up of more than 100 organisations: Governmental and non‐governmental funders (1) award money to research projects. Academic publishers (2) provide infrastructure for the review, curation, dissemination and promotion of findings by publishing them in their journals (3). Repositories (4) store and help curate various research outputs providing attribution to data and code, and often enable free and immediate access to these forms of information to share and reuse. Advocacy organisations (5) support and lobby for certain aspects of open research, such as through the Center for Open Science, cOAlition S and the Research Data Alliance (RDA). Many learned societies (6) are leaders in organising conferences and generating networks that are vital to disseminating knowledge, supporting researchers and facilitating collaboration (e.g. Figure 1). Societies are also involved in publishing in partnerships with publishers. Researchers are embedded within this landscape, but are not included within the analyses as researchers lack a mission and vision statement.

We explore trade‐offs and the for‐/not‐for‐profit divide across the research landscape (Figure 1) to identify which stakeholders are most and least aligned with open research and how this distribution impacts the mobilisation of knowledge. Alignment with open research principles is quantified from several text‐based analyses of the mission and vision statements of 129 stakeholder organisations from six stakeholder groups. We use mission and vision statements as reflections of strategic planning, highlighting a stakeholders' motives, objectives and values, which could align or conflict with one another and the open research agenda. These mission and vision statements are linked to stakeholders from across ecology and evolutionary biology, and thus tied to global challenges like the biodiversity crisis. We hypothesise that stakeholder group's alignment with open research principles and their business models (e.g. for vs. not‐for‐profit) drive the distribution of conflicts and trade‐offs in the landscape, creating barriers to the fast deployment of open research. Our objective is to pinpoint where in the research landscape changes can be made to accelerate knowledge mobilisation that supports research and policy development.

## 2 MATERIALS AND METHODS

To identify differing priorities and trade‐offs between and within stakeholders, we use three text analysis approaches (Topic Detection, Theme Exploration and Dictionary analysis—see below) to detect commonalities and differences in the mission and vision statements (*N* = 129) from six stakeholder groups in the research landscape. Here, we present an overview of our methods; a comprehensive description of our methods is available in the form of a webpage and annotated Rmarkdown documents at the following link: https://andbeck.github.io/workflowr‐policy‐landscape/.

Our stakeholder groups include: Journals (*N* = 30; 16 are open access)—highest impact ecology and evolution journals from https://www.scimagojr.com/; Publishers (*N* = 14; 6 are for‐profit)—journal publishers; Funders (*N* = 30)—international and national research funders, with a limit of up to three national funders per country; Repositories (*N* = 17)—online archives for data and code; Advocates (*N* = 24)—a group of organisations that actively support or promote good quality and accessible research; and Societies (*N* = 13)—organisations to promote networking and collaboration among within the academy. Our stakeholder groups are unevenly distributed (e.g. naturally, there are going to be more journals than publishers), and many of the stakeholders we identified are not solely limited to the field of ecology and evolution (e.g. publishers often have a portfolio of journals across different fields). We have specifically searched for stakeholders from across the world, however, we have only used stakeholders with English mission and vision statements. This limits our findings solely to the English research landscape.

We did not contact anyone associated with the stakeholders to request more information about their aims, missions, visions or goals. If their website did not have a dedicated section but the missions or visions were described in the ‘About’ section, this was deemed an acceptable proxy. In some cases, the stakeholder public websites lacked anything resembling a mission or vision statement, in which case, we removed the stakeholder from our sample. In very few cases, a stakeholder was involved in more than one group (e.g. The Royal Society as a society and a publisher); here, we collected both statements and treated them as a separate stakeholder. All of our stakeholder statements were compiled in August 2021. The specific date the statements were published online is unknown. Prior to any text analyses, we first cleaned the text following the recommended steps in Maier et al. (2018).

### 2.1 Topic detection

To characterise the differing priorities between stakeholders, we first identified the key topics of discussion within the mission and vision statements. To do this, we used a mixed‐modelling approach to combine quantitative structural topic modelling (Roberts et al., 2019) and qualitative grounded theory (Corbin & Strauss, 1990). Topic modelling describes the unsupervised process of identifying common clustering of words within documents (in our case, sentences). Essentially, if the words ‘open’ and ‘research’ are regularly used together, they will likely form the basis of a cluster (topic). Topics are then attributed to each document, so each sentence in each mission or vision statement is assigned a topic. We used the structural topic modelling approach as this permits covariates to be captured within the topic modelling. This was advantageous for our data as it allowed us to capture the data's hierarchical nature, specifying two factorial covariates: where each sentence belonged to a particular mission or vision statement, and one of the six stakeholders.

An important parameter within topic modelling is selecting how many topics to produce; a variety of approaches have been developed for selecting topic frequency (Greene et al., 2014). We used the Mimno and Lee (2014) approach, which automates selection of topic frequency by using t‐SNE algorithm—which has been shown to outperform comparative approaches (van der Maaten & Hinton, 2008). This automated topic number selection is advantageous as it avoids modellers having to subjectively select their own number of topics. However, a potential downside is that the automated approach can lead to a large number of topics being selected—in our case, we found 73 topics—and these topics can often co‐vary, for example, topic 1 could be more similar to topic 2 than topic 3.

To handle the large number of topics, we used a commonly applied qualitative approach called grounded theory to consolidate the large number of topics down into core topics of discussion in the vision and mission statements. Grounded theory is not only designed to develop concepts and aid interpretation of unstructured data sources like text (Corbin & Strauss, 1990) but is also valuable for interpreting topics. Grounded theory has three core steps:

Open coding—Where we characterised each topic according to common and influential words. For instance, if the words ‘open’, ‘research’ and ‘access’, were common, we used our intuition and understanding of the word meanings to describe these as words related to ‘research being openly accessible’.
Axial coding—After developing open codes for each topic, we looked for similarities and links between each of the open coded topics. For instance, open codes of ‘broadly related to the process of peer review’ and ‘submitting work to journals’ would be linked.
Selective coding—After identifying links between the topics, we conceptualised these linked topics into selective codes, for example, groups of topics, which we call themes from this point onwards. For instance, the axial links above would fall into our theme of ‘Publishing process’.

We developed four main themes, which we define as: Open research—sentences related to making research, data or software openly accessible, with an ethos of transparency and accountability; Community & Support—focussed on fostering relationships between stakeholders and researchers, or between researchers themselves; Innovation & Solution—based around using research to derive impact and solve problems; Publication Process—focussed around the practical steps needed to publish work. We then re‐labelled each sentence with their respective theme, that is, if topic 1 was placed in the ‘Publication process’ theme, all sentences with topic 1 were converted to ‘Publication process’. Finally, to report the differing priorities between the stakeholder groups, we assessed the proportional frequency of each theme in each mission statement. We report the mean and 50% quantiles across the mission statements for each stakeholder in each topic.

Grounded theory is designed to be explorative (i.e. things should not be coded strictly based‐off a predefined schema). However, as our research question is primarily focussed around open research and trade‐offs in the research landscape, with clear hypotheses, we opted to ignore distinctly irrelevant topics, placing them into an additional theme: ‘Other’. All ‘Other’ sentences were removed from the analysis.

### 2.2 Theme exploration

To further explore the themes, we look at which words are most common within the stakeholder groups and assess word associations, that is, words likely to appear in a sentence together. Word frequencies offer insight into the primary focus of the text, and associations add context. For instance, if words related to the ‘Open research’ and ‘Innovation & Solution’ themes were common and regularly associated with each other, we could speculate that this stakeholder considers these themes linked, where perhaps open research is a pathway to innovation. In this analysis, we removed words that appear in less than 5% of statements and only kept the top 10% most‐common words, assessed at the stakeholder group level. We visualise word frequency and associations using hierarchical edge bundling, a dendrogram clustering approach designed to simplify word associations, a form of adjacency relation.

### 2.3 Dictionary analysis

Our final text analysis approach is designed to determine which stakeholder groups favoured business‐ and open research‐based language. To do so, we compiled multiple dictionaries for business terms (i.e. words related to business; our expectation is that profit focussed stakeholder groups will use more business‐based language), and compiled them into one common dictionary—see https://andbeck.github.io/workflowr‐policy‐landscape/. We were unable to locate a dictionary of open research words, so instead we created one from the recent UNESCO recommendation on Open Science (UNESCO, 2021). As both dictionaries were variable in size and had repeated words, we opted to identify 100 words for each dictionary that we considered a good representation of business and open research language. To do this, we calculated word frequencies in each dictionary, and ranked both dictionaries by these words, whereby the most common words occurred at the top of the list. We then extracted the 100 most common words in each dictionary and compared each of our mission statements to these dictionaries, that is, what proportion of words each statement occurs in each dictionary. We report the mean proportion and 50% quantiles across each stakeholder group.

We use these three text analysis approaches to compare differences between stakeholders, but following reviewer feedback it was apparent that comparative analyses were also needed within stakeholders. Within the results, we begin highlighting differences between stakeholders which each have different profit motivations, for example, for‐profit (publishers, journals) and not‐for‐profit (advocates, funders, repositories, societies). Then we address issues around for‐ and not‐for profit stakeholders at two scales by focusing on publishers and journals. First, we divide publishers and journals into groups: for‐profit (*N* = 6) and not‐for‐profit (*N* = 8) publishers and OA (*N* = 16) and non‐OA (*N* = 14) journals. Second, we divide journals into for‐profit OA journals (*N* = 8), for‐profit non‐OA (*N* = 11), not‐for‐profit OA (*N* = 6) and not‐for‐profit non‐OA (*N* = 5) journals. We conduct the same language analysis on the subgroups that were conducted on the main stakeholder groups.

## 3 RESULTS

### 3.1 Open research is a low priority for half of the stakeholders

**FIGURE 2:**
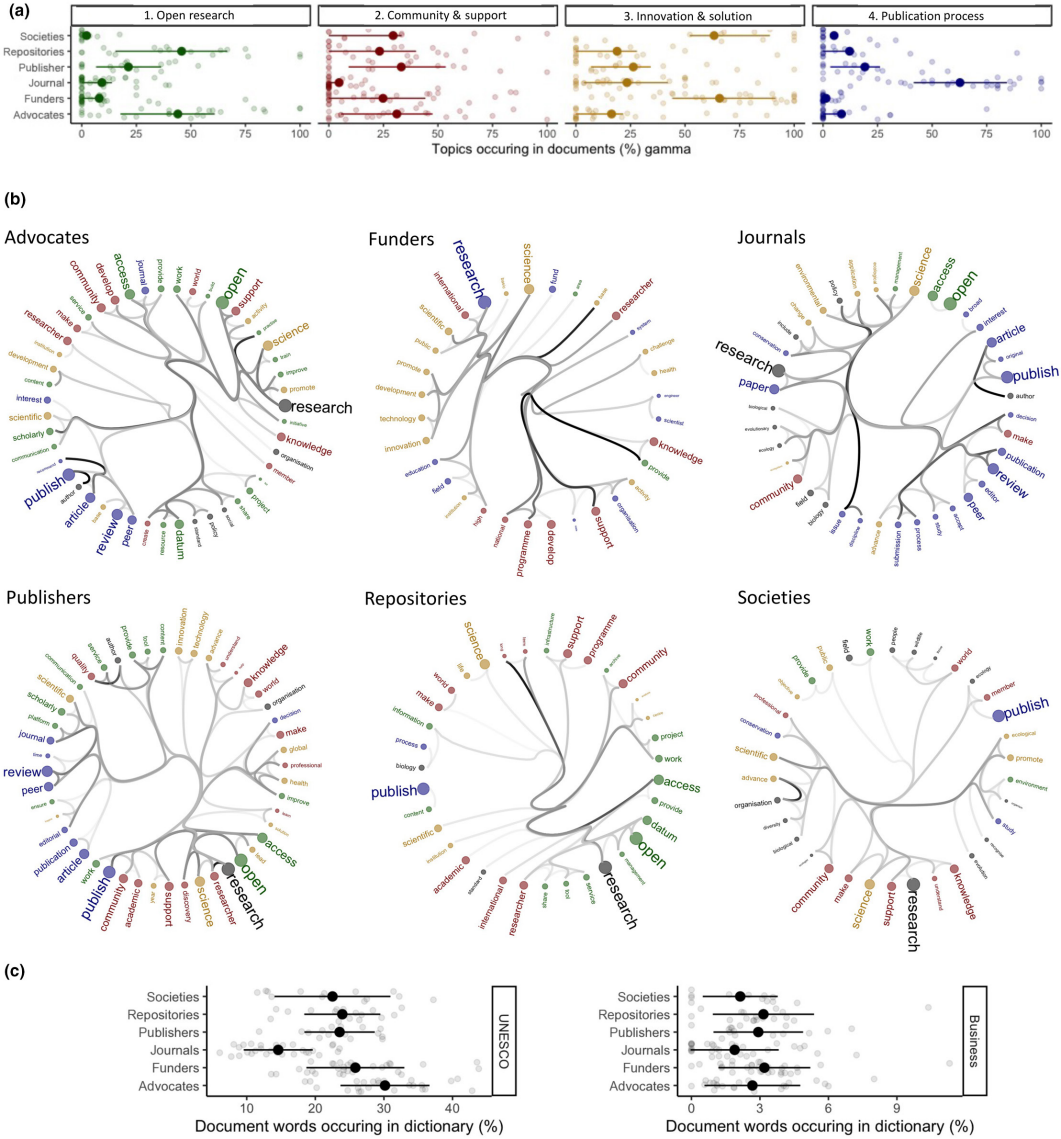
Stakeholders have different commitments. Application of text analyses to define topics (a), reveal word‐associations within stakeholders (b) and explore business and open research language use between stakeholders (c). (a) We detected four main topics (side labels): Open research (green), Community and Support (red), Innovation and Solutions (yellow) and Publication process (blue). Gamma values (*x*‐axis, means ± SD) are the strength of association between a set of stakeholder documents (*y*‐axis) and the corresponding topic. High gamma values are strong associations. (b) Hierarchical edge bundling networks of word associations aligned with topics for each stakeholder group. All words are coloured by their primary topic and strong (thick lines) links among words define the core vocabulary for each stakeholder. (c) Stakeholders have stronger associations on average with UNESCO (2021) vocabulary than business language. Journals have the weakest and advocates the strongest association with UNESCO. Publishers have neither the strongest nor weakest association with UNESCO or business vocabulary.

Journals (9.19%), funders (7.97%) and societies (2.16%) have very low levels of open research language in their mission statements (Figure 2a). Figure 2b confirms these patterns highlighting that the strategies of journals, funders and societies are dominated by publishing process and community support language, but not open research vocabulary.

*Journals*' strategies appear disproportionately focused on the publication process (Figure 2a, plot 4 and Figure 2b, colour = blue) over open research or even community and support (Figure 2a, plots 1 and 2). Journals' mission and vision statements had the lowest percentage of shared vocabulary with UNESCO (Figure 2c). *Societies* have the weakest alignment with the open research topic but a strong alignment with innovation and community support (Figure 2a,b, network = societies). They have a moderate association with UNESCO vocabulary (Figure 2c). *Funders* and *societies* also reflect more focus on innovation and solutions (Figure 2a, plot 1). Funders and societies do differ however, with funders exhibiting high connectance between the innovation and solution vocabulary and the community support vocabulary. In contrast, societies have low connectance among these types of vocabulary (Figure 2b).

### 3.2 Where is open research best represented?

*Repositories* and *advocacy* group mission and vision statements show the strongest alignment with open research, community and support and innovation and solution topics (Figure 2a,b). For the repository stakeholder group, open research terms are also highly connected to the community and support topic terms (Figure 2b; Repositories).

In contrast to current dogma, *publishers*' mission and vision statements are not misaligned with the open research topic or UNESCO vocabulary (Figure 2a–c). They are the third most associated with the open research topic, the strongest association with community and support and contain moderate representation of all topics and vocabulary, including UNESCO's (Figure 2a–c).

### 3.3 Business language and profit model

Business language in the statements, as a proxy for ‘for‐profit’ constraints, did not distinguish among stakeholder groups (Figure 2c). These data reveal no substantive difference between for‐profit (publishers, journals) and not‐for‐profit (advocates, repositories, funders) stakeholders. Our dictionary may not be refined enough to detect such differences or business vocabulary is just not as common a defining strategy as we hypothesised.

### 3.4 The same roles, different business models

There is a dogma within the community that publishers and journals are ultimately stifling research progress with profit focussed business models. However, publishers and journals are diverse entities within themselves, with varied open access policies and profit motives. Here, focussing first on publishers, we compare language use between for‐ and not‐for‐profit publishers. Then, we compare language use between OA and non‐OA journals. Finally, offering particular scrutiny towards journals, we assess language use across four types: not‐for‐profit OA journals, not‐for‐profit non‐OA journals, for‐profit OA journals and not‐for‐profit non‐OA journals. This comparison falls at the crux of the debate around open research, where not‐for‐profit OA journals are rightly expected to foster open research, especially compared to for‐profit journals. Similarly, we would anticipate a high use of business vocabulary in for‐profit non‐OA journals as these journals typically require payment or a subscription to access full‐text articles and the apparent goal of for‐profit journals is to generate revenue and make a profit.

### 3.5 For‐profit versus not‐for‐profit publishers

**FIGURE 3:**
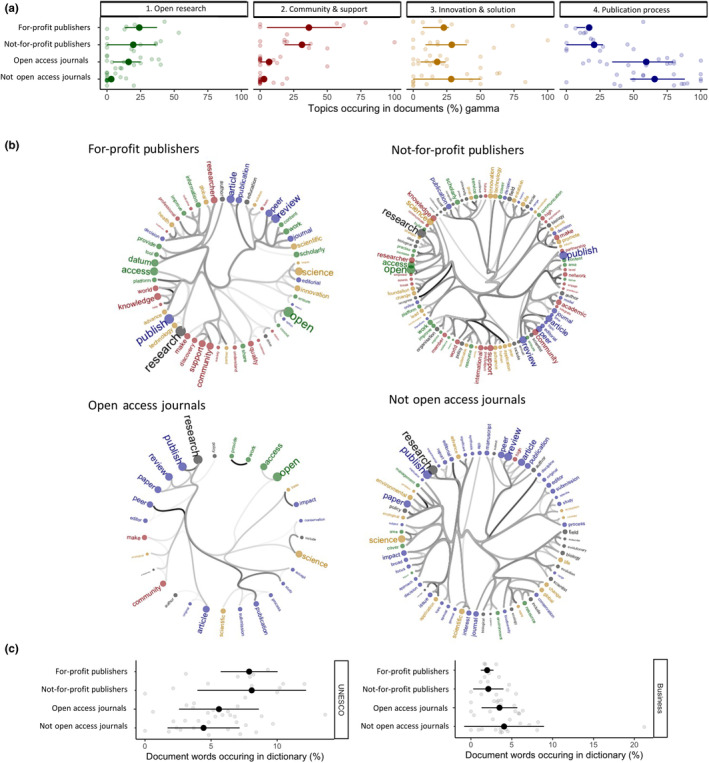
Commitments of publishers and journals are similar despite different business models. Application of text analyses to define topics (a), reveal word‐associations within subgroups of chosen stakeholders (b) and explore business and open research language use between these groups (c). (a) We looked at the alignment of for‐profit and not‐for‐profit publishers, and OA and non‐OA journals with four main topics (side labels): Open research (green), Community and Support (red), Innovation and Solutions (yellow) and Publication Process (blue). Gamma values (*x*‐axis, means ± SD) are the strength of association between a set of stakeholder documents (*y*‐axis) and the corresponding topic. High gamma values are strong associations. (b) Hierarchical edge bundling networks of word associations aligned with topics for each stakeholder group. All words are coloured by their primary topic and strong (thick lines) links among words define the core vocabulary for each stakeholder. (c) Neither of the groups associate more with UNESCO or business vocabulary. Business language is also low in all groups.

We expected to see that for‐profit publishers would show a lower alignment with open research principles in their mission and vision statements and reflect a higher use of business vocabulary. Contrary to what we anticipated, there is no difference in topic alignment between the for‐profit and not‐for‐profit publishers' mission and vision statements and their alignment with all the topics including open research. They seem to similarly align across all topics (Figure 3a) with publishers generally showing the strongest association with community and support. Additionally, for‐profit and not‐for‐profit publishers show a comparable language pattern to UNESCO (Figure 3c, plot 1) and there is no clear statistically significant distinction between the two groups. Furthermore, when considering the usage of business vocabulary (Figure 3, plot 2) there is no discernible difference between for‐profit and not‐for‐profit publishers.

### 3.6 Open access policies in journals

We expected to observe a higher alignment with the open research principles in OA journals compared to non‐OA journals because Open Access is a relevant component of the open research agenda. Following our expectations, Open Access journals align more with the open research topic compared to non‐OA journals (Figure 3a, plot 1). However, it is apparent within the word connections from the text similarity analysis (Figure 3b, Open Access journals) that the high alignment with open research is driven primarily by the presence of two words—open and access—rather than a sign of commitment towards open research. There is no significant difference between the OA and non‐OA journals in their alignment to the remaining topics (Figure 3a, plots 2–4). Both groups of journals are similarly more focused on the publication process (Figure 3a, plot 4 and Figure 3a, colour = blue) rather than open research or community and support (Figure 3a, plots 1 and 2). Furthermore, when conducting language analysis, it showed that both OA and not‐OA journals share a similar number of vocabularies with UNESCO (Figure 3c, plot 1), and there is no clear difference between the groups. There is also no difference in the use of business vocabulary between these journals' groups (Figure 3c, plot 2).

### 3.7 Profit in OA and non‐OA journals

**FIGURE 4:**
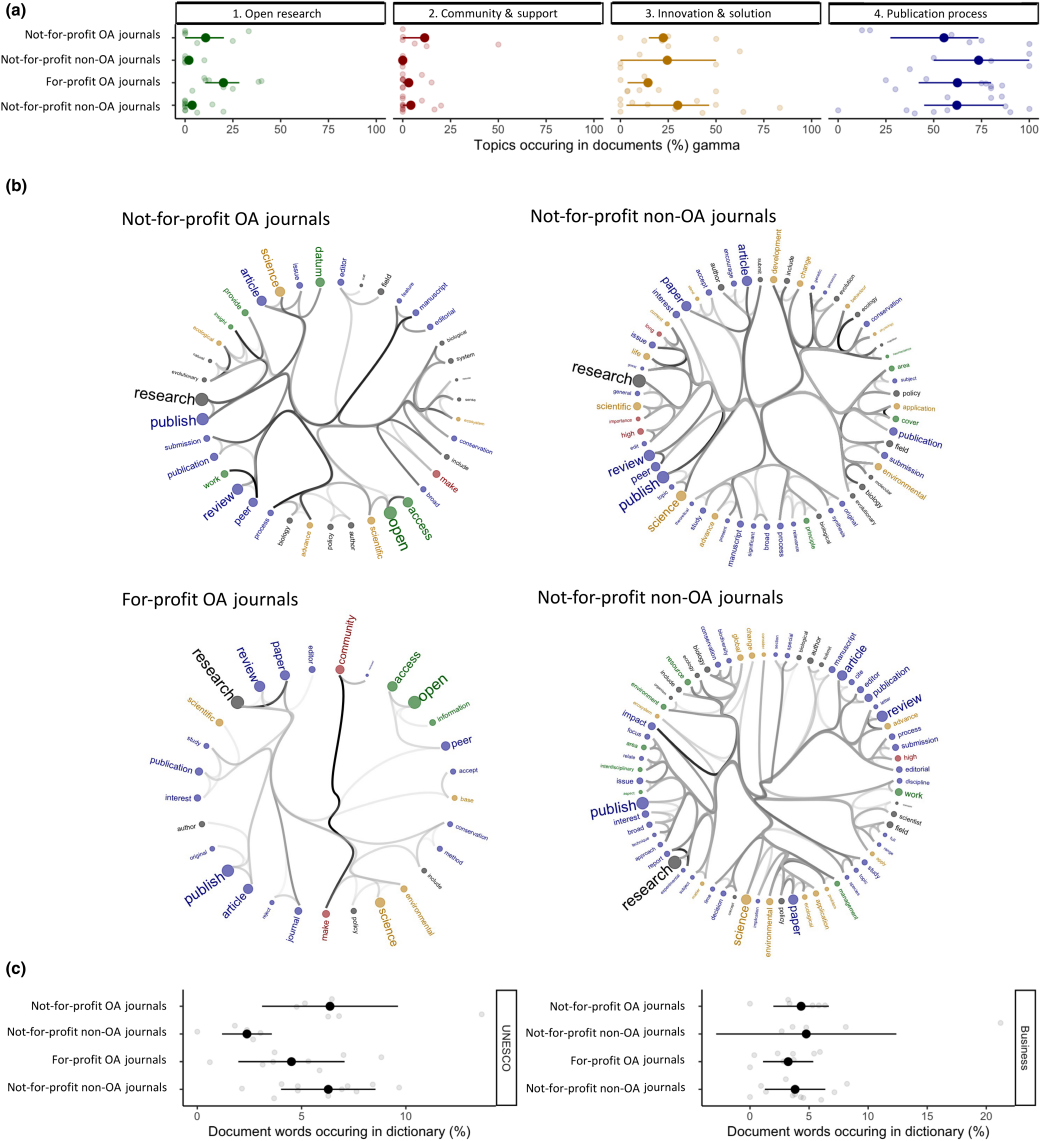
Commitments of journals are similar despite differing business models and OA (open access) policies. Application of text analyses to define topics (a), reveal word‐associations within subgroups of chosen stakeholders (b), and explore business and open research language use between these groups (c). (a) We looked at the alignment of for‐profit OA and non‐OA, and not‐for‐profit OA and not‐OA journals with four main topics (side labels): Open research (green), Community and Support (red), Innovation and Solutions (yellow) and Publication Process (blue). Gamma values (*x*‐axis, means ± SD) are the strength of association between a set of stakeholder documents (*y*‐axis) and the corresponding topic. High gamma values are strong associations. (b) Hierarchical edge bundling networks of word associations aligned with topics for each stakeholder group. All words are coloured by their primary topic and strong (thick lines) links among words define the core vocabulary for each stakeholder. (c) Neither of the groups associate more with UNESCO or business vocabulary. Business language is also low in all of these groups as well.

In contrast to our expectations, not‐for‐profit OA and for‐profit OA journals' mission and vision statements similarly align with the open research topic (Figure 4, plot 1), with all journal types favouring innovation and solution (Figure 4a, plot 3) and publication process (Figure 4a, plot 4). Additionally, all four journal types share similar proportions of UNESCO vocabularies (Figure 4c, plot 1) and business vocabularies (Figure 4c, plot 2). It is worth noting, within these comparisons, we class journals published solely by learned societies as not‐for‐profit.

## 4 CONCLUSIONS AND RECOMMENDATIONS

Our analyses revealed that journals, funders and societies (half of stakeholders) lack alignment in their mission and vision statements with open research vocabulary suggesting low priority in strategic planning. They may not have accommodated open research principles in strategic planning, might lack an understanding of the benefits open research can provide or do not deem it necessary to show support and discuss their commitments towards the open research agenda. Here we focus on four additional insights.

First, the *for‐profit* or *not‐for‐profit* dogma that publishers present a barrier to open research is not represented in our mission and vision statements (Eddy, 2019; Larivière et al., 2015) or in vocabulary tied to business and profit (Figures 2c and 3c). Instead, *publishers* are part of 50% of the stakeholders (with advocates and repositories) that use more open research language (Figure 2a–c), potentially representing an awareness in strategic planning of the opportunities and threats to publishing associated with open research. More detailed analyses of publishers' alignment with the open research topics or use of business vocabulary (Figure 3) indicate that this is irrespective of for‐profit and not‐for‐profit status of publishers (Figure 3c). Furthermore, there is ample evidence that for‐profit publishers are accelerating the transformation of their subscription journals into Open Access (Eddy, 2019) and strengthening their recommendations around archiving and sharing data in appropriate public repositories (Sholler et al., 2019). Many of these recent transitions align with guidelines encouraging researchers to publish their manuscripts open access and deposit data following FAIR principles; guidelines linked to big funders driving global initiatives such as Plan S (Eddy, 2019) and the new US government policy on immediate public access to tax‐payer funded research by 2025 (Brainard & Kaiser, 2022).

Second, *journals* appear to be weakly aligned with open research, community & support, and innovation & solution. Instead, journals' statements almost exclusively focus on the process researchers use to publish rather than the benefits to readers and users of information (Figure 1, Issues 4–6). This introduces a discrepancy, where the missions and visions set out by publishers (which have a relatively even distribution across topics) fail to propagate into journals.

One source of the discrepancy between journals and publishers might be a result of the changing policies created by funders and governments. The topics related to open research identified in publishers' statements, may come from publishers adapting to a changing landscape towards more transparent and open research. This adaptation may simply not have filtered through to journals yet, given the rapid pace of change in the research landscape. In this scenario, a misalignment between publishers and journals may purely represent a communication gap.

An alternative and purely speculative hypothesis for misalignment between publishers and journals, could be that the public promotion of the open research agenda by publishers may poorly represent their true commitments, which may instead align with profit‐focussed business models, that is, open‐washing. We have no evidence to state that this is the case though, and find it somewhat unlikely given open research does not inherently prevent publishers profiting, that is, through article processing charges.

One potential reason for the mismatch between publishers and journals could purely be an artefact of the stakeholders targeting different audiences. Journals focus on authors, while publishers make their profile more visible inside and outside academia. This is perhaps why we identified a stronger commitment from publishers across all topics, especially to community support and innovation (Figure 2a), whereas journals provide practical information related to peer‐review, submission and the publication process (Figure 2b, Journals). This explanation appears particularly likely given open research language is comparable between the four core journal types: not‐for‐profit OA journals, not‐for‐profit non‐OA journals, for‐profit OA journals and not‐for‐profit non‐OA journals (Figure 4). Although the absence of an effect—we expected higher open research language use in not‐for‐profit OA journals than for‐profit non‐OA journals—could also simply be a type 2 error, where we failed to detect an effect due to a small sample size. More work would be needed to resolve whether this absence of an effect is genuine or not.

It is perhaps logical that journals focus on highlighting the publication process. More often than not, journal strategy is set by academic societies and publishers, and focused on author experience. However, journal's editorial boards and their author/readership play a crucial role in shaping researchers' conduct through the establishment of policies and serve as guardians of academic standards. We suggest that it is vital that open research ideals increase prominence in journal mission and vision statements. Recognising this potential can not only enhance the journals' impact but also fortify their position as influential agents guiding towards more transparent and open research landscape.

Regardless of how open research language is used, it is important for the research landscape to remain cautious, that in the transition to open access, one form of inequity—where subscription paywalls prevented a researcher from accessing research—is changed for another, where APC barriers prohibit researchers from publishing their research. Unaffordable APC charges are not in line with the open research strategy that many organisations advocate for and have even led to mass resignations of editorial boards (Sanderson, 2023). Thus, a transition from a subscription journal model to an open access model could be part of a strategic decision to maintain current business models rather than shift to a model more aligned with open research philosophies. This conjecture about the business models may be reflected in the poor record of transition from standard and hybrid to open access by two major for‐profit publishers, Springer Nature and Elsevier who failed to meet their open access transition targets in Plan S (Silver, 2023).

The mission and vision statements of many *societies* and *funders* are also very weakly aligned with open research principles (Figure 1, Issues 1 & 2). This is a missed opportunity because both stakeholders have a platform to engage simultaneously with researchers and publishers directly and encourage best open research practices that support the transition towards a more open and transparent landscape. However, the focus on innovation and community in these stakeholders may indirectly represent open research as these topics may be tightly connected (Besançon et al., 2021). Alternately, with societies often reliant on sharing the proceeds of publisher income (Brainard, 2019), societies may feel financially constrained from adopting open research principles despite publishers using open research language.

Finally, as expected, the mission and vision statements of *advocates* and *repositories* possess some of the highest associations with the open research topic and vocabulary, as well as high references to other topics (Figure 1, Success 1 & 2). These organisations have entered with an explicit agenda of commitment and support for values and language cared for by other stakeholders, providing infrastructure, all while recognising the importance of supporting the community and open research principles.

### 4.1 From pattern to action

Against these insights, Figure 1 summarises the locations of key issues, barriers and successes. To ensure the mobilisation of research and data to support policy, more stakeholders in the open research landscape need to take a more formal and interactive stand for undisturbed knowledge flow. Here we offer several recommendations that could facilitate such changes.

The main concentration of barriers can be seen around the journals and publishers, even though publishers themselves are more aligned than others. We suggest this nexus reflects low collaboration between stakeholders and limited alignment with the open research agenda by the journals. Our analyses suggest benefits will arise from journals pivoting their mission and strategy away from process and more towards alignment with open research. This shift to focus less on authors per se and more on facilitating links between authors and recipients of knowledge and data will capitalise on the agenda and passion in the academic community represented by societies, editorial boards and authors.

Funders by their policies, and societies via collaboration and meetings, might further lead this transition towards a more open research landscape. Funder links with governments represent opportunities to define guidelines and standards, establish incentives and develop a culture where institutions and researchers find it easy to pivot towards open research. Societies, as representatives of the researcher who are tasked with mobilising data, might increase engagement with funders to facilitate rapid uptake of open research standards. Advocates can support this by further defining high‐quality standards and with funders lowering financial and administrative barriers for researchers to increase openness.

There are immense opportunities across the entire landscape to improve collaboration and communication. While some of this is already in place (e.g. links between repositories and advocates; Figure 1, see tick boxes), to truly accelerate open research, more agreements among stakeholder groups are needed. This will ultimately avoid wasted resources and ensure that the financial and administrative burdens on researchers is lowered and journals position as the conduit to policy is opened; this collaboration must extend the impact of science to businesses, policy and non‐governmental organisations.

Tackling global challenges requires an agile research landscape that can deliver trusted and robust warning signals and solutions to drive evidence‐based policies (Gauchat, 2015). The severity and rapid acceleration of many global challenges require substantial increases in the reporting, publishing and sharing of knowledge and best practices beyond the research landscape to the network of people and organisations developing policy. There is no single solution to bridge this gap but removing barriers to adopting open research principles is a clear first step. Free and easy access to the most up‐to‐date trustworthy research, and reducing the burden on researchers of making this happen, should be a priority for all stakeholders in face of the current emergencies.

## AUTHOR CONTRIBUTIONS

**Zuzanna B. Zagrodzka:** Conceptualization (equal); data curation (lead); formal analysis (lead); funding acquisition (equal); methodology (equal); software (equal); visualization (lead); writing – original draft (lead); writing – review and editing (lead). **Thomas F. Johnson:** Formal analysis (supporting); methodology (supporting); supervision (supporting); writing – original draft (supporting); writing – review and editing (equal). **Andrew P. Beckerman:** Conceptualization (equal); funding acquisition (supporting); project administration (lead); supervision (lead); writing – original draft (supporting); writing – review and editing (equal).

## CONFLICT OF INTEREST STATEMENT

ZBZ is funded by Jisc and UK Reproducibility Network. She is a member of the Sheffield Reproducibility Network, the Sheffield Open Research Working Group and a former member of SORTEE. APB is on the Board of Directors at DRYAD and an Editor‐in‐Chief at Ecology and Evolution. TFJ is a former member of SORTEE.

## Data Availability

A comprehensive description and implementation of our methods is available in the form of an open source/access webpage and annotated Rmarkdown documents at the following link: https://andbeck.github.io/workflowr‐policy‐landscape/.
